# Hookworm treatment induces a decrease of suppressive regulatory T cell associated with a Th2 inflammatory response

**DOI:** 10.1371/journal.pone.0252921

**Published:** 2021-06-10

**Authors:** Virginie Doyen, Francis Corazza, Hoa Nhu Thi, Thanh Le Chi, Carine Truyens, Carole Nagant, Hiep Tran Thi Mong, Jean-Francois Fils, Phuong Thi Ngoc Huynh, Olivier Michel

**Affiliations:** 1 Laboratory of Translational Research, ULB223, CHU Brugmann, Université Libre de Bruxelles (ULB), Brussels, Belgium; 2 Clinic of Immunoallergology, CHU Brugmann, ULB, Brussels, Belgium; 3 Immunology Laboratory, LHUB-ULB, Brussels, Belgium; 4 Parasitology and Mycology Department, Pham Ngoc Thach University of Medicine, Ho Chi Minh, Vietnam; 5 Immunology Laboratory, Pasteur Institute, Ho Chi Minh, Vietnam; 6 Parasitology Laboratory, ULB Center for Research in immunology (U-CRI), Université Libre de Bruxelles, Brussels, Belgium; 7 Department of Family Medicine, Pham Ngoc Thach University of Medicine, Ho Chi Minh, Vietnam; 8 Ars Statistica SPRL, Boulevard des Archers, Nivelles, Belgium; 9 Department of Hematology, Bordet Institute, ULB, Brussels, Belgium; UMASS Medical School, UNITED STATES

## Abstract

**Background:**

Like other helminths, hookworms (HW) induce a regulatory immune response able to modulate and dampen reactivity of the host to antigens. No data about the evolution of the immune response after treatment are available. We aim to phenotype the regulatory immune response during natural HW infection and its evolution after treatment.

**Methodology:**

Twenty hookworm infected (HW+) and 14 non-infected subjects HW–from endemic area in the periphery of Ho Chi Minh City were included. Blood and feces samples were obtained before, 2 and 4 weeks after treatment with Albendazole 400mg. Additional samples were obtained at 3 and 12 months in the HW+ group. Hematological parameters, Treg (CD4+CD25^hi^FoxP3^hi^) and surface molecules (CD39, CD62L, ICOS, PD-1, CD45RA) were measured as well as inflammatory and lymphocytes differentiation cytokines such as IL-1β, IL-6, IFNγ, IL-4, IL-17, IL-10, IL-2 and TGFβ.

**Results:**

HW+ subjects showed higher Treg, TregICOS+, Treg PD1-, TregCD62L+ and CD45RA+FoxP3^lo^ resting Treg (rTreg). CD45RA-FoxP3^lo^ non-suppressive Treg cells were also increased. No preferential Th1/Th2 orientation was observed, nor difference for IL-10 between two groups. After treatment, Treg, TregICOS+, TregCD62L+, Treg PD1- and rTreg decreased while IL-4 and IL-6 cytokines increased.

**Conclusion:**

During HW infection, Treg are increased and characterized by a heterogeneous population: a highly suppressive as well as a non-suppressive T cells phenotype. After treatment, Treg with immune-suppressive phenotype exhibited a decrease parallel to an inflammatory Th2 response.

## 1. Introduction

Helminths are pluricellular organisms that can induce prolonged infection by modulating the immune host response. An equilibrium settles between the parasite and the host due to mechanisms that have co-evolved for thousands of years. Among helminths, infection with hookworms (*Necator americanus*, *Ancylostoma duodenale or seldom Ancylostoma ceylanicum)* (HW) is one of the most prevalent neglected tropical diseases and represents a paradigm of manipulation of the host’s immune system by a parasite. In most cases, the host immune response fails to remove adult worms from the bowel, and switches to a regulatory phenotype that enables the HW to colonize the gut for many years [1, 2].

Exploring the mechanisms underlying the state of immune tolerance occurring during chronic helminth infection may provide an interesting approach to develop new treatment based on immunomodulation for allergic and autoimmune diseases. Experimental animal models exist, but they do not accurately mimic the course of human infection [2]. In human, HW infection has been associated with an increase in regulatory T cells (Treg), upregulation of regulatory cytokines such as interleukin 10 (IL-10) and transforming growth factor beta (TGFβ), an unbalanced Th1/Th2 response and a higher baseline level of tumor necrosis factor [3–5]. From mouse models, we know that molecules from A. caninum induced Treg that are able to protect from allergic asthma and to control the proliferation of mouse and human effector T cells *in vitro* [6].

Treg is a highly immunosuppressive subpopulation of CD4+ T cells, characterized by transcription factor forkhead box P3 (Foxp3). They play a crucial role in the maintenance of immune homeostasis and self-tolerance [7]. However, they are a heterogeneous population and they exerts their function through different cellular and soluble mechanisms. In 2009, Miyara et al. demonstered that human Treg are composed of 3 phenotypic subpopulations based on expression of CD45RA: the resting (CD45RA+FoxP3^lo^, rTreg), activated (CD45RA−FoxP3^hi^, aTreg) and cytokines producing non-suppressor Treg cells (CD45RA−FoxP3^lo^). They showed that they exert distinct suppressive capacities [8, 9]. Other molecules such as ectonucleoside triphosphate diphosphohydrolase 1 (E-NTPDase1, CD39), cell adhesion (CD62L) and checkpoint molecules (Inducible T cell co-stimulator, ICOS), Programmed cell death 1, PD1) are associated with higher Treg immune-suppressive functions. The CD39 converts ATP (a danger signal that may activate inflammasome) into AMP that will be metabolized by CD73 into adenosine and inhibit T cell activation [10, 11]. L-selectin (CD62L) is a type I transmembrane glycoprotein and cell adhesion molecule that has been shown to be associated with a higher immunosuppressive capacity and a better chemokine-driven migration to secondary lymphoid organs [12–15]. ICOS (CD278) is highly expressed on Treg [16] and the ICOS signaling pathway endows Tregs with increased generation, proliferation, survival and high suppressive T cells abilities [17, 18]. PD-1 is a type I transmembrane protein that belongs to the immunoglobulin superfamily. PD-1 bears the immunoreceptor tyrosine-based inhibitory motif in its cytoplasmic region and acts as a negative regulator of immune responses [19, 20]. Expression of IL-2R (CD25) that is a characteristic of Treg play an important role in suppressive Treg function by inhibiting the production of IL-2 through a cell contact dependent manner [21]. The suppression of CD4^+^ T cell activities by Tregs is also mediated by inhibitory cytokines including transforming growth factor β (TGF-β) and IL-10, the latter being important for its immunosuppressive activity at environmental interfaces [22, 23].

The aim of this study was first, to characterize the Treg phenotype during chronic HW infection and after its treatment and secondly, the Th1/Th2 balance and inflammatory responses.

## 2. Methods

### 2.1. Study population

The study has been conducted in Hoc Mon (between 08/2014 to 03/2016), a rural region at 10 kilometers from the center of Ho Chi Minh City, where HW is an endemic helminthiasis. People were recruited by the local health center among 50 volunteers that were screened, and subjects may be considered as representing a larger local population. Twenty volunteers aged 18–65 years old, infected with HW (HW+), were recruited. Diagnosis of HW infection was based on analysis of duplicate Kato-Katz thick smear slides. The slides were examined under a microscope by experienced laboratory technicians. Parasite load was expressed as number of eggs/g of feces [24]. Other intestinal helminth infections (*Strongyloides stercoralis and Ascaris lumbricoides*) were excluded. Exclusion criteria were pregnancy, a positive history of allergic and autoimmune diseases or human immunodeficiency virus (HIV), and a previous anti-helminthic treatment during the past 6 months. Fourteen non-infected healthy controls (HW-) living in the same area were included.

### 2.2. Study design

At inclusion (T0), medical history, anthropomorphic, and the type of household were recorded, and all subjects received 1 dose of 400 mg Albendazol. Follow-up visits were scheduled at 2 weeks (2 W) and 1 month (1 M). An additional follow up visit was performed 3 and 12 M after treatment for some participants of the HW+ group, but not for the control group. At each visit, clinical examination was performed, and blood samples (8mL in heparinized tubes for hemogram analysis and PBMC separation, and 4mL in serum separator tubes) and feces for Kato–Katz and direct examination were obtained. The study was approved by the Ethics Committee (EC) of Pham Ngoc Thach University (Ho Chi Minh City, Vietnam, IRB-VN01013) and was conducted in accordance with all applicable regulatory requirements and Good Clinical Practice. The ethics statement was amended by the Brugmann EC (Number 2021/72). Each participant provided signed informed consent prior to enrollment in the study. The study has been registered on clinical.trials.gov (NCT02262403).

### 2.3. Standard biological parameters

Hemogram, albumin and ferritin were measured. Serology was tested against *Ascaris lumbricoides* (Ouchterlony immunodiffusion test with home-made Ascaris extract). The antigen is coelomic fluid collected from adult *A*. *suum* worms (obtained from the intestines of infected pigs at a local abattoir), used at a concentration of 2 mg freeze dryed fluid /10 μL. Patient sera were concentrated 3 fold by freeze-drying before used. A positive control was used, consisting of hyper-immune serum of a rabbit immunized against the antigen. Diffusion was performed on agarose gels (1.3% Indubiose A37—MP Biomedical, Illkirch-Graffenstaden, France, in Veronal buffer pH 7.4 –Westburg, Leusden, NL). After 48h, gels were incubated for 2h in Na citrate 5% in water to eliminate potential unspecific precipitation bands due to reaction between serum CRP and carbohydrate residues present in the antigen preparation. Gels were then washed twice during 2h in NaCl 9% to eliminate unprecipitated proteins, demineralized for 1h in distilled water and dried. They were then stained with Amido black 10B 0.12% in acetate buffer 1M to visualize precipitation bands.

The *Toxocara canis* and *Strongyloides stercoralis* serologies were tested by ELISA (antibodies detection kit from IVD Research, Inc, IVD TC-96 and IVD STRONGY-96, Carlsbad, CA, USA; sample dilution 1/64).

### 2.4. Flow cytometry

PBMCs were isolated from sodium heparinized plasma by gradient density centrifugation on Lymphoprep® washed and then re-suspended in RPMI-1640 medium supplemented with 10% fetal bovine serum (RPMI-FBS). To characterize Treg, a total of fresh 10^5^ cells were stained with CD4, CD25, CD45RA, CD39, CD62L and CD4, CD25, PD1, and ICOS and incubated in the dark for 30 minutes. After fixation and permeabilization steps with the FoxP3 buffer set, FoxP3 was stained with an anti-FoxP3-Alexa antibody (S1 Table). For Treg, the gating strategy was based on the side scatter/forward scatter lymphocyte, CD4+, CD25^high^, and FoxP3+ populations. At least 15000 CD4 were acquired. To discriminate Treg populations positive and negative for ICOS and PD-1, gates were fixed on the lymphocyte population. Next, Treg were separated in 3 groups based on the expression of CD45RA and FoxP3. The following subpopulations were defined: rTreg (CD45RA+ FoxP3^lo^), aTreg (CD45RA-FoxP3^hi^) and non-suppressive T cells (CD45RA-FoxP3^lo^) [8]. All antibodies and flow cytometry reagents were bought from BD Biosciences (BD Biosciences, San Jose, CA, USA). Measurements were performed using the BD FacsCanto flow cytometer and analyzed using FlowJo X 10.0.7r2 software (Tree Star, Inc.).

### 2.5. Cytokines serum levels

Multiplex cytokine measurements (IL-4, IL-2, IFNγ, IL-17, IL-10, TGF-β, IL-6, and IL-1β) were performed using a plate-based electrochemiluminescence assay according to manufacturer’s instructions (MSD, Meso Scale Discovery, Rockville, MD, USA; catalog numbers: K151A0H-2, K151XWK-1, K151A9H-2, K151A3S). The lower limits of detection (LoD) were respectively 0.54 fg/mL, 0.09 pg/mL, 0.37 pg/mL, 0.4 pg/mL, 0.04 pg/mL, 9.1 pg/mL, 0.06 pg/mL, 0.05 pg/mL.

### 2.6. Statistical analysis

Statistical analysis was performed using the software R (R Core Team, 2019, version 3.6.2). For baseline characteristics and demographic data, continuous data were compared by means of T-test when homogeneity of variances, tested with the Bartlett’s test, and normality of the residuals, tested with the Shapiro-Wilks test, were reached, and means and standard deviations (means ± SD) are reported. When homogeneity of the variance or normality of the residuals were not proved, Wilcoxon signed rank test was performed on rank data and medians and inter-quartile ranges (median [Q25 –Q75]) are reported. For count data, the Pearson Chi-Squared test was performed to compare proportions.

For variables measured several times in patients, a linear mixed model [25] was used to model the evolution of biological variables through time.

The next effects will be tested: a group effect (HW), a time effect (T) and a group * time (HW*T) interaction effect meaning that: 1) we will test whether groups have different level for the studied variable (= group effect); 2) there is an evolution (increase or decrease) of the studied variable through time (= Time effect); 3) groups will be tested whether they have a different evolution of the studied variable through time (time*group interaction effect).

To compare data at 3M and 12M available only for the HW+ group, the time and time-square effects were tested. A positive time effect indicates that the parameter increases as time goes by and a negative time square that the parameter follows an initial increase before a decrease at the end of the study (and inversely). If the residuals of the model are not normally distributed, we will use the bestNormalize R package to transform the outcome and report the results of this last linear mixed model. Maximum Likelihood (ML) uses all available data in the study, produces unbiased estimates of the treatment effect and correct p-values. Correlation were tested with a Pearson correlation test at T0 in the HW+ group. A p value < 0.05 was considered statistically significant (*p < 0.05, **p < 0.01, ***p < 0.001).

## 3. Results & discussion

### 3.1. Population (S2 Table)

Despite the World Health Organization’s (WHO’s) deworming strategy for controlling infection and reducing the worm burden, HW is still prevalent in Vietnam with an estimated prevalence of 25% and large variation within the country [26–28]. Between 2014 and 2016, 20 HW-infected subjects and 14 controls were evaluated before and after treatment. Several subjects were lost in both groups (9 in the HW+ and 1 in the HW- group) due to a poor adherence and 4 subjects were re-infected with HW in the HW+ group and were excluded from the statistical analysis. Both groups were comparable in terms of age, sex ratio, usual medications, and home environmental characteristics, with the exception of the Body Mass Index and as expected, occupation and the presence of a garden (S2 Table) [1, 29]. The burden of infection in the HW+ group (240 [48–1584] egg/g feces [median with range]) according to World Health Organization (WHO) criteria was low [30]. This low intensity of infection is in accordance with data observed in other countries [31], with the relatively low prevalence of HW infection in this population [32] and hypothetical former deworming treatments [33]. Serologies were negative for *A*. *lumbricoides* and *S*. *stercoralis*, confirming the direct examination results. Some subjects (4 HW- and 1 HW+) had a positive serology against *T*. *canis*. Seroprevalence of toxocariasis was estimated to be 45.2% in Ho Chi Minh City [34] and is due to cultural habits that expose people to *Toxocara* eggs through contamination of environment with infected dog’s feces [35]. The proportion of positive serology for *Toxocara* was well balanced between both groups, no clinically significant toxocariasis was observed and the antiparasitic treatment was not sufficient to cure toxocariasis. Moreover, positive serology might result from antigenic cross-reactions or previous infections [34]. Consequently, it should not affect our results.

### 3.2. Standard biological results (S3 Table)

As expected, higher eosinophils and lower neutrophils (%) was observed in the HW+ group (p<0.05). Eosinophils level was slightly increased in the HW infected group. This may be related to the low intensity of infection [36, 37] and the chronicity of infection. Indeed, it has been shown that the levels of eosinophils spontaneously decrease after migration of the larval form [38]. Moreover, some HW- subjects also exhibited an increased level of eosinophils, due to *Toxocara*-infected subjects. The treatment of infection induced a decrease of eosinophils in percentage (p < 0.05) and in absolute value (p<0.05). No anemia or hypoalbuminemia was observed in our population suggesting that subjects did not suffer from overt inflammatory enteritis. It is in accordance with the low intensity of infection [39]. The treatment of infection induced a transient increase of the albumin level (p<0.01) in the HW+ group.

### 3.3. Immunological parameters

#### 3.3.1. Regulatory T cells populations

**Fig 1:**
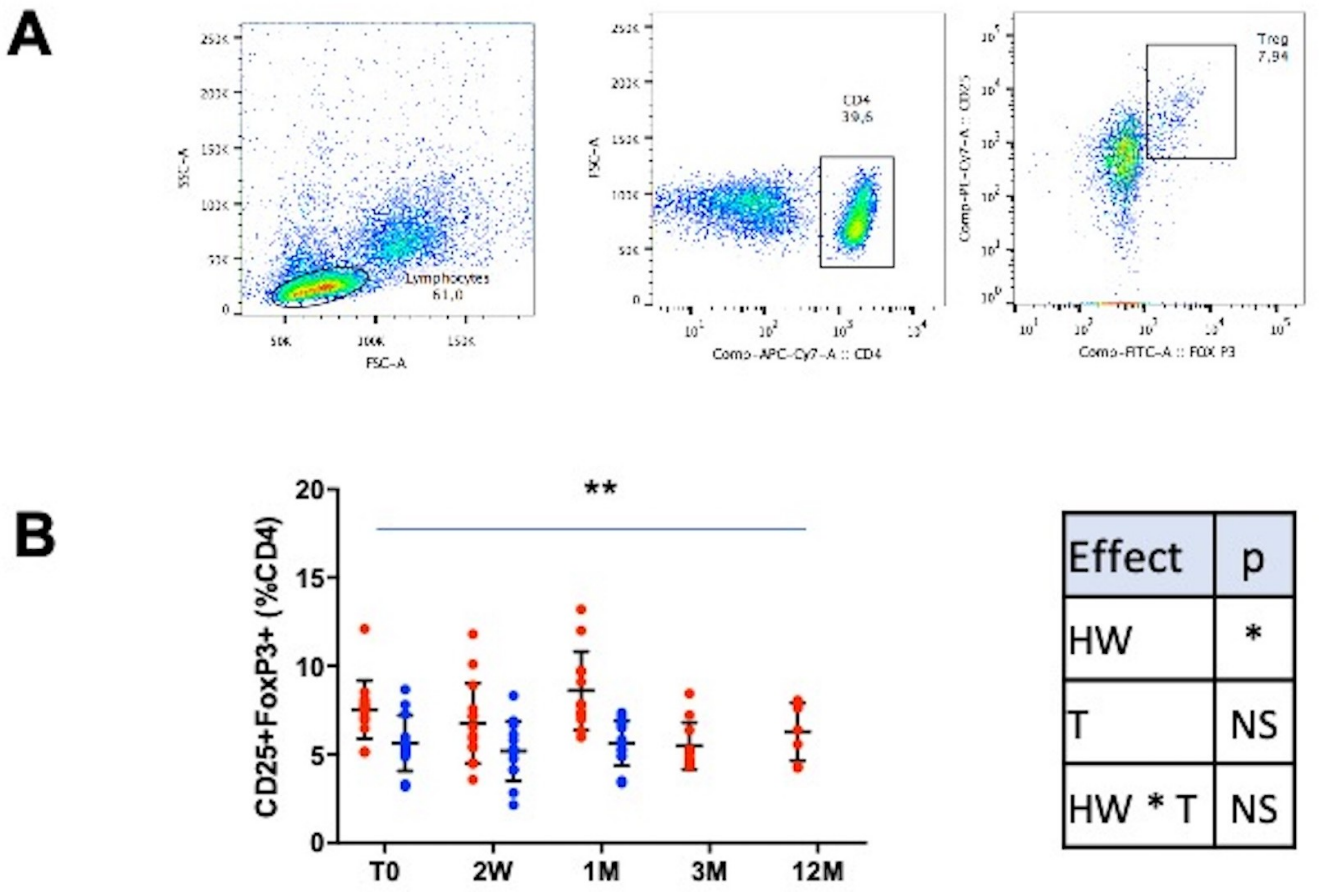
**Treg.** A: Gating strategy. B: Results are expressed as mean +/- SD. HW+: population infected with hookworm, HW-: control group, 2W: 2 weeks, 1M: 1 month, 3M: 3 months and 12M: 12 months after treatment. The HW+ group is represented with red dots and the HW- group with blue dots. To compare the HW+ and HW- groups and their evolution, a maximum likelihood analysis has been used to test a group (HW), a time (T) and a group * time (HW * T) interaction effects (with data at T0, 2W and 1M). Statistical results are summarized in correspondent tables for each parameter. To compare longitudinal data from the HW+ group (T0, 2W, 1M, 3M and 12M), a time and a time^2^ effects were tested. The time effect is depicted on the figures and the time^2^ effect is given in the results section (* p<0.05, ** p<0.01, ***p<0.001).

*3.3.1.1. Treg (Fig 1).* HW+ subjects showed a higher percentage of CD4+CD25^hi^FoxP3^hi^ Treg compared to HW- (p < 0.05). The treatment of the infection induced a decrease of Treg (p<0.01) with a positive time^2^ effect (p<0.05). In literature, the causal links between helminth infection and Treg have been well established. During helminthiasis, Treg are more numerous and active, and decrease after treatment [40]. Treg activation is essential for the parasites to survive [41]. Our study confirmed the results from Ricci et al. reporting a higher Treg level in a HW+ Brazilian population [5]. This study also showed that mononuclear cells from infected subjects had a lower proliferative capacity but didn’t looked at the surface’s molecules nor evolution after treatment.

**Fig 2:**
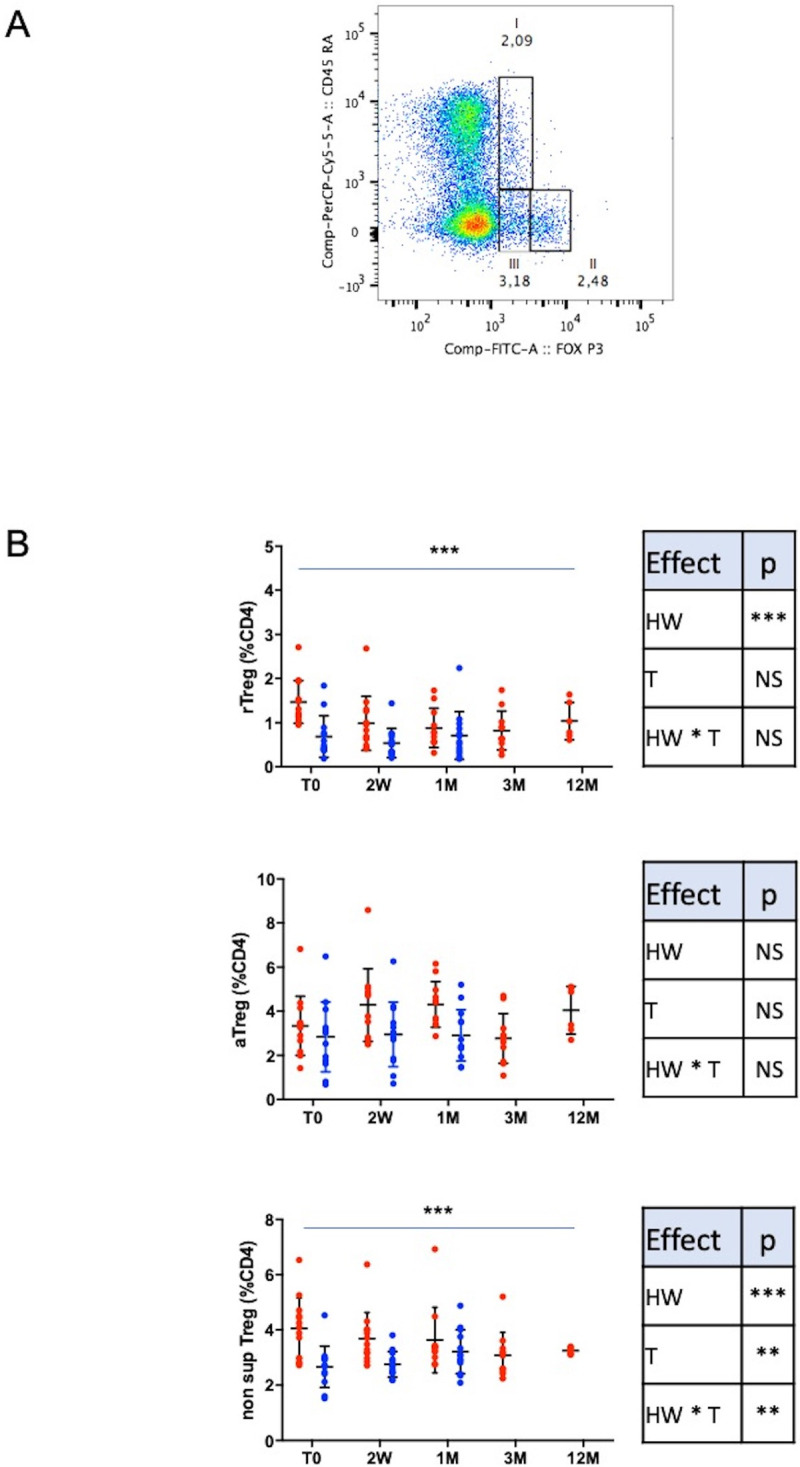
Treg sub-populations. A: Example of gating strategy to define the 3 subpopulations of Treg. CD45RA+FoxP3^lo^ (rTreg), CD45RA-FoxP3^lo^ (non-suppressive T cells) and CD45RA-FoxP3^hi^ (aTreg). B: Longitudinal results of rTreg, a Treg and non-suppressive Treg in the HW+ et HW- groups. Results are expressed as mean +/- SD. HW+: population infected with hookworm, HW-: control group, 2W: 2 weeks, 1M: 1 month, 3M: 3 months and 12M: 12 months after treatment. The HW+ group is represented with red dots and the HW- group with blue dots. To compare the HW+ and HW- groups and their evolution, a maximum likelihood analysis has been used to test a group (HW), a time (T) and a group * time (HW * T) interaction effects (with data at T0, 2W and 1M). Statistical results are summarized in correspondent tables for each parameter. To compare longitudinal data from the HW+ group (T0, 2W, 1M, 3M and 12M), a time and a time^2^ effects were tested. The time effect is depicted on the figures and the time^2^ effect is given in the results section (* p<0.05, ** p<0.01, ***p<0.001).

*3.3.1.2. Treg sub-populations based on CD45RA and FoxP3 expression (Fig 2).* According to CD45RA and FoxP3 expressions, Treg were categorized into three phenotypic and functionally distinct subpopulations: CD45RA+Foxp3^lo^ (rTreg), CD45RA^−^Foxp3^hi^ (aTreg), and CD45RA^−^Foxp3^lo^ Treg (non-suppressive Treg) [7, 8]. The gating strategy is depicted Fig 2A. Compared to HW-, the HW+ group expressed higher levels of rTreg (p < 0.001) and non-suppressive Treg cells (p < 0.001). The treatment of HW infection induced an early decrease of both rTreg and non-suppressive Treg (p<0.001) with positive time^2^ effect (p<0.01). Activated Treg (aTreg) were not different between both groups (Fig 2B). The treatment of HW infection induced an early decrease of both resting Treg (rTreg) and non-suppressive Treg, but not for activated Treg (aTreg). Usually, we believe that aTreg is the major subset of suppressive Treg and plays a more important role in the regulation of immune response. The results found in this study may trigger re-investigation of the distinct suppressive ability of these Treg subtypes during HW chronic infection. In other model such as chronic hepatitis B infection it has been shown that mTreg, the mouse equivalent of aTreg, increase during the inflammatory phase. Indeed, mTreg possess pro-inflammatory chemokine receptors and could migrate to the inflamed tissue and inhibit the immune responses [42].

We observed a higher level of rTreg during HW infection. No comparative data are available. In other models such as HIV, rTreg numbers were preserved [9] and in transplantation, they were associated with better outcomes [43, 44]. We also showed an increase in non-suppressive Treg cells during HW infection. Literature revealed that those non-suppressive Treg cells had a high capacity to produce IL-17 as well as high of IL-2 and IFNγ under stimulated conditions [8].

**Fig 3:**
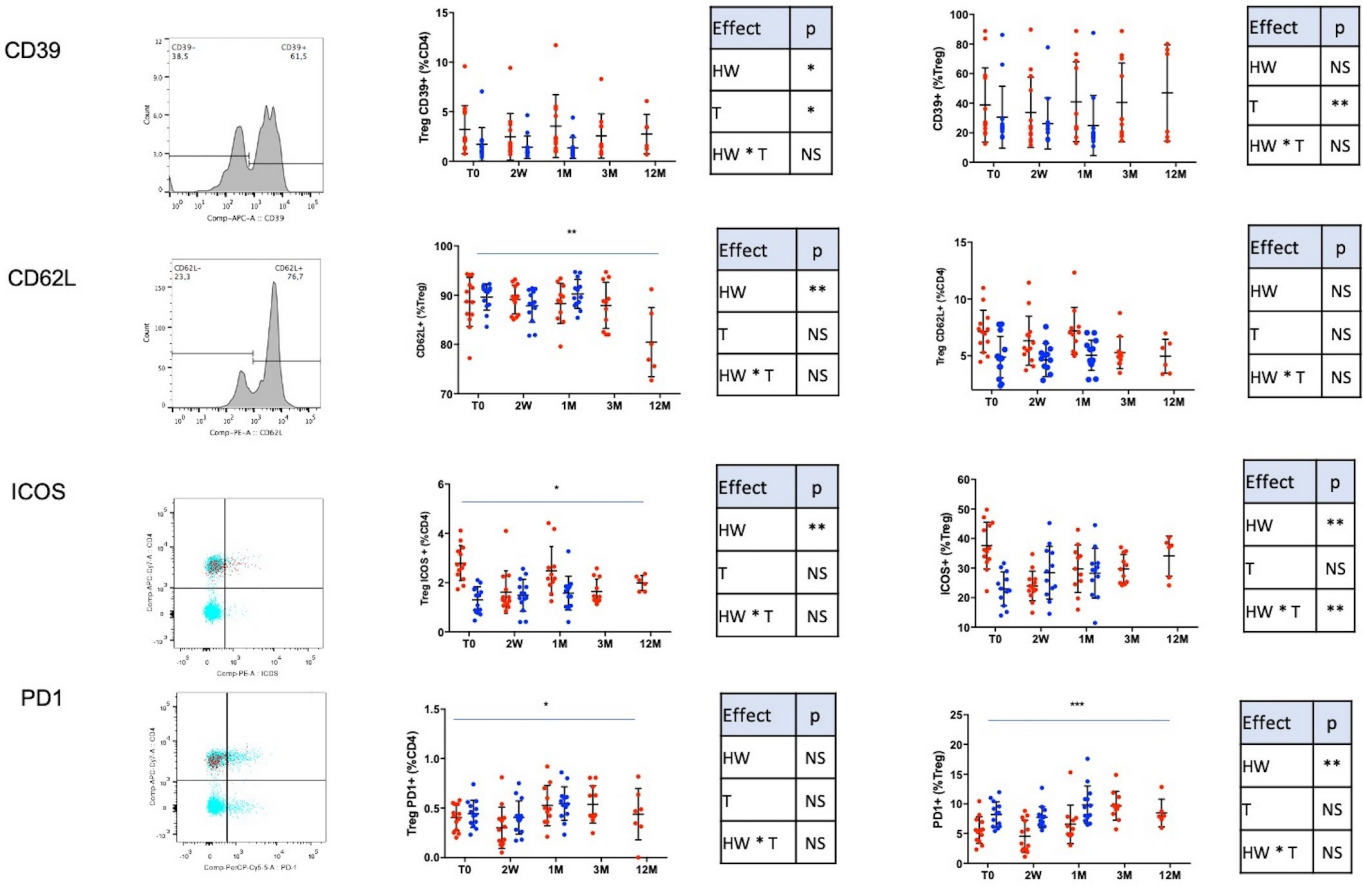
Treg function markers. Results are expressed as mean +/- SD. HW+: population infected with hookworm, HW-: control group, 2W: 2 weeks, 1M: 1 month, 3M: 3 months and 12M: 12 months after treatment. The HW+ group is represented with red dots and the HW- group with blue dots. Fig 3 depicted CD39 (%Treg) and Treg CD39+ (%CD4); CD62L (%Treg) and Treg CD62L+ (%CD4); costimulatory molecules ICOS(%Treg) and TregICOS, and PD-1 (%Treg) and TregPD1 (%CD4). To compare the HW+ and HW- groups and their evolution, a maximum likelihood analysis has been used to test a group (HW), a time (T) and a group * time (HW * T) interaction effects (with data at T0, 2W and 1M). Statistical results are summarized in correspondent tables for each parameter. To compare longitudinal data from the HW+ group (T0, 2W, 1M, 3M and 12M), a time and a time^2^ effects were tested. The time effect is depicted on the figures and the time^2^ effect is given in the results section (* p<0.05, ** p<0.01, ***p<0.001).

*3.3.1.3. Suppressive phenotype (Fig 3).* 3.3.1.3.1. E-NTPDase1 (CD39). Ectonucleoside triphosphate diphosphohydrolase 1 (E-NTPDase1), also known as CD39 is expressed on a subset of highly suppressive Treg. Levels of CD25+FoxP3+CD39+ (%CD4) were higher in HW+ group (p<0.05). An early significant decrease after treatment was observed in both groups. No significant change was observed after 1 year of follow up. Expression of CD39 on Treg was not significantly different between HW+ and HW-. No other data during HW infection are available. In a microfilaria model, Lima et al. [45] demonstrated that a higher proportion of CD4+ cells expressed CD39.

3.3.1.3.2. Selectine-L (CD62L). Our results showed a higher level of CD25^high^FoxP3^high^CD62L+ (%CD4, p<0.01) in the HW+ and a decrease after treatment (p<0.01). The percentage of CD62L (%Treg) was not different among groups and no change occurred after treatment. L-selectin (CD62L) correspond to a naïve phenotype confirming the evolution of the rTreg [12–14].

3.3.1.3.3. Checkpoint molecules (ICOS, PD1). The HW+ group exhibited more CD25^hi^FoxP3^hi^ICOS+ (Treg ICOS+, %CD4, p<0.01) compared to HW- group. Treg ICOS (%CD4) showed a significant decrease after treatment (p<0.05) with a positive time^2^ effect (p<0.05) indicating a global decrease with an increase trend of Treg ICOS at the end of the study. More Treg expressed ICOS (%) in the HW+ group (p < 0.01). The interaction effect was significant, indicating a decrease of ICOS+ (%Treg) during the first month after the treatment (p<0.01) in HW+. Data on ICOS during helminth infection is available in a mouse model, showing that ICOS plays a key role in driving Treg expansion and function by regulating type 2 immunity towards helminths [46]. In allergy and asthma, other Th2 cell-mediated diseases, ICOS influences also the control of the number of developing Th2 clones [47, 48]. Our results showed that HW infection is associated with higher Treg ICOS as well as ICOS positive Treg and that ICOS expression quickly decreased after treatment.

Programmed cell death 1 (PD-1, CD80) is another important checkpoint molecule that acts as a negative regulator of immune responses [19, 20]. In our study, Treg PD1+ (%CD4) were not different among 2 groups but they showed an increase in the HW+ group after the treatment (p< 0.05) (with—time^2^ effect, p<0.05). Fewer Treg expressed PD-1 in HW+ compared to HW- (p < 0.01). The treatment induced an increase of PD1 (%Treg, p<0.001) through time (with a negative time^2^ effect; p < 0.001). Our results are in accordance with those from Raimondi et al. showing that Treg with suppressive activity did not express surface PD-1 [44]. Moreover, it has been showed that low PD-1 Treg has a higher capacity to elicit B cell apoptosis and inhibit CD4 T cells [43]. In our study, less Treg expressed PD-1 during HW infection and levels increased once the infection is cured to the level similar than HW-. It suggests a highly suppressive phenotype during HW infection. Moreover, Treg PD-1 results are in accordance with the higher level of Treg CD62L+ and rTreg which exhibited naïve phenotype [49].

#### 3.3.2. Activated CD4

Activated CD4 population (CD25^hi^FoxP3-) was also increased in HW+ compared to HW- (p < 0.001) (S1 Fig). The treatment induced a rapid (<0.001) and persistent decrease throughout the study (with a + time^2^ effect (<0.001)). It is in accordance with other data showing that T cells (CD4, CD8) as well as B cells exhibited an activated profile during HW infection [4]. Moreover, Treg and CD4+CD25^hi^FoxP3- were positively correlated in the HW+ group (r 0.282, p<0.05) as well as in the all population study (r 0.35, p<0.001) indicating that mechanisms behind the induction of those populations are probably linked.

#### 3.3.3. Cytokines (Fig 4)

Lymphocytes Th1/Th2 differentiation cytokines such as IFNγ, IL-4 were not different between HW+ and HW- groups. However, the level of IL-4 was lower in HW+ if we increased the error alpha at 10% (p = 0.059) and the treatment induced an IL-4 increased (p<0.05) (negative time^2^ effect, p<0.05). The absence of Th2 preferential differentiation may be surprising. However, we have to consider that our model is a chronic natural infection combining different stage of larval cycle that is known to be associated with a mixed Th1/Th2 response [50, 51].

**Fig 4:**
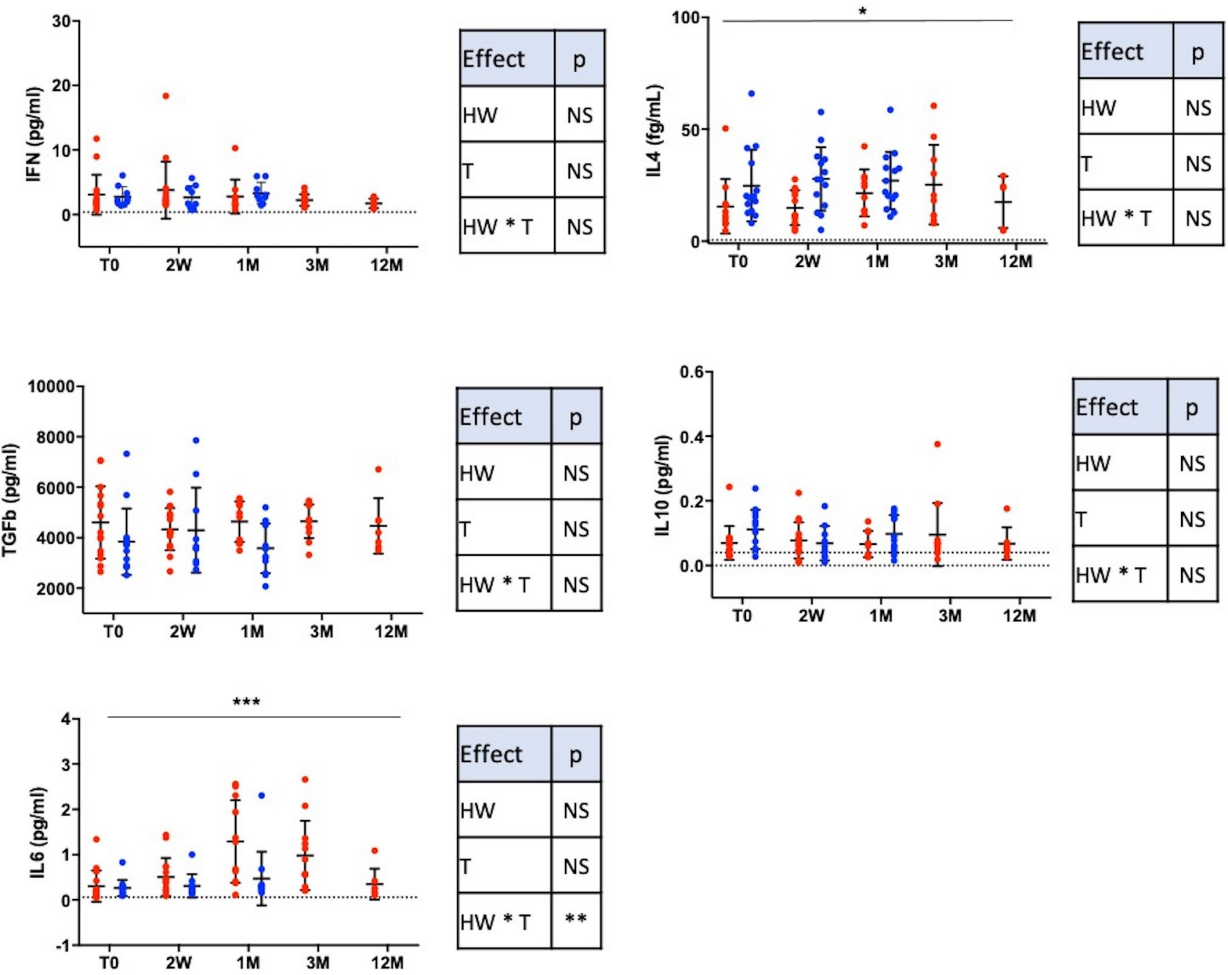
Systemic cytokines. Results are expressed as mean +/- SD. 2W: 2 weeks, 1M: 1 month, 3M: 3 months and 12M: 12 months after treatment. Lower limit of Detection (LoD) is symbolized with dotted line. The HW+ group is represented with red dots and the HW- group with blue dots. To compare the HW+ and HW- groups and their evolution, a maximum likelihood analysis has been used to test a group (HW), a time (T) and a group * time (HW * T) interaction effects (with data at T0, 2W and 1M). Statistical results are summarized in correspondent tables for each parameter. To compare longitudinal data from the HW+ group (T0, 2W, 1M, 3M and 12M), a time and a time^2^ effects were tested. The time effect is depicted on the figures and the time^2^ effect is given in the results section (* p<0.05, ** p<0.01, ***p<0.001).

After treatment, we observed an increase of IL-4. This may be of importance taking into account the role of IL-4 in the IgE isotypic switch and may be a hypothesis to explain the increase of allergic sensitization after treatment of HW disease.

No difference among HW+ and HW- groups was observed for TGF-β or IL-10 nor effect of the treatment. This observation was surprising as IL-10 is one of mode of action of Treg. However, our results are in accordance with Geiger et al. who showed that IL-10 secretion was significantly diminished in stimulated PBMCs from infected individuals and that IL-10 production were stage-specific [4, 52]. The stage of infection was impossible to precise in our natural infection settings. Moreover, the major contributors to IL-10 production are FoxP3neg Tr1-like cells that we did not measure in our study [53].

IL-6 increased after the treatment of infection (group-time effect p<0.01 and longitudinal analysis p<0.001). It may be due to the inflammatory response induced by parasitic death and release of damaged cellular components. In the context of allergic diseases, it has been shown that Il-6 played a pathological role in asthma model and in the priming of individual mast cells for subsequent IgE-mediated activation [54, 55]. Among cytokines, IFN was positively correlated with Treg (%CD4) (r = 0.75 **). Other cytokines such as IL-2, IL-17 and IL-1β were not analyzed because below the LoD.

Taking together, treatment of HW infection induce persistent changes of significant Treg and soluble parameters. However, for some of them, we observed an inverse trend at the end of the study for those parameters (represented by a time^2^ effect). It may be linked to silent reinfections (subjects were still exposed to infection risk factors) but also to a decrease in the subject’s number due to lost subjects.

In this study, we characterized the Treg and systemic cytokine profile of HW infection (before and after treatment) in comparison with HW-. We chose a chronic infection model in an endemic area. However, this approach was complicated by local constraints of a developing country in a tropical region leading to some limitations of this study. First, the small number and the loss of subjects as well as the disequilibrium between HW+ and HW- groups which required specific statistical analysis in the context of a longitudinal study. Second, the co-infection of some subjects. Third, other factors may have interfered with our results such as the microbiota and the intestinal barrier function. The gut microbiota plays an important role in the extrathymic differentiation of Treg, where short-chain fatty acids produced by commensal microorganisms, facilitates generation of Treg [56]. Some data suggest that helminth infection can modulate microbial dysbiosis and impacts elements of the epithelial barrier, which influence the immune regulatory status [57–60].

This study has also some strengths that are the well characterized population (general and biological characteristics), the fact that the study was based on natural chronic infected population as well as the follow up during several months.

## 4. Conclusion

Taken together, our results confirmed previous studies showing an increase of Treg during chronic HW infection [5]. However, we demonstrate in this study that Treg are composed by a mixed population. On one hand, a phenotype associated with high immunosuppressive capacities such as high levels of ICOS+ and a naïve Treg profile (CD62L+, PD-1- and CD45RA+) and on the other hand, a non-suppressive T cells and activated CD4 population. This observation has also been described in a filarial model [59]. This is combined with systemic cytokines levels suggesting an absence of overt inflammation without preferential Th1/Th2 orientation. Some suppressive phenotypes, such as CD39, CD62L, are not related to the HW infection. The treatment of infection was associated with a decrease in Treg level and a switch in their phenotype, namely, a decreased expression of ICOS and of naïve Treg and a systemic IL-6 and IL-4 inflammatory response.

Those results may be put into perspective with allergic diseases that occur more frequently after treatment of helminthiasis. Indeed, the decrease of highly suppressive Treg, the transient IL-6 inflammatory response concomitant with IL-4 increase after treatment of the parasitic disease are immunological factors that may contribute to a Type 2 inflammatory response favoring allergic diseases as observed in epidemiological data [61].

## Supporting information

S1 Fig Activated CD4+CD25^hi^FoxP3- lymphocytes. HW+: hookworm infected subjects, HW-: control subjects. 2W: 2 weeks, 1M: 1 month, 3M: 3 months and 12M: 12 months after treatment. HW+ group is represented with black square with continuous lines and HW- group with a blank circle with discontinuous lines. To compare the HW+ and HW- groups and their evolution, a maximum likelihood analysis has been used to test a group (HW), a time (T) and a group * time (HW * T) interaction effects (with data at T0, 2W and 1M). Statistical results are summarized in correspondent tables for each parameter. To compare longitudinal data from the HW+ group (T0, 2W, 1M, 3M and 12M), a time and a time^2^ effects were tested. The time effect is depicted on the figures and the time^2^ effect is given in the results section (* p<0.05, ** p<0.01, ***p<0.001).
(DOCX)

S1 Table Antibodies used in flow cytometry experiments. (DOCX)

S2 Table Population characteristics. (✻): Medical history: HW+: stroke, steatosis 2, hypertension, hepatitis B; HW-: hypertension 3, digestive bleeding, dengue, lithiasis, valvulopathy, Basedow disease. Comparison of proportion was tested with Pearson Chi-square test and comparison of mean with t-test. (* p<0.05, ** p<0.01, ***p<0.001).
(DOCX)

S3 Table Standard biological results. Results are expressed as mean +/- standard deviation. WBC: white blood count, N: Neutrophils, Eo: Eosinophils, Ly: Lymphocytes, Hb: Hemoglobin, MCV: Mean corpuscular volume, Alb: Albumin, NS: Non-significant. Maximum likelihood (ML) analysis on data from the 2 groups (at T0, 2W and 1M) were performed: a group effect, a time effect and a group * time interaction effect were tested. The time and time-square effects were tested on the longitudinal date from the HW+ group (ML 1 group). A positive time effect indicates an increase throughout the study and negative time-squared effect a decreased at the end of the study (and inversely).
(DOCX)

## References

[1] Loukas A, Hotez PJ, Diemert D, Yazdanbakhsh M, McCarthy JS, Correa-Oliveira R et al. Hookworm infection. Nat Rev Dis Primers. 2016 Dec 8;2:16088. doi: 10.1038/nrdp.2016.88 27929101

[2] Gaze S, Bethony JM, Periago MV. Immunology of experimental and natural human hookworm infection. Parasite Immunol. 2014 Aug;36(8):358–66. doi: 10.1111/pim.12088 25337625

[3] Geiger SM, Massara CL, Bethony J, Soboslay PT, Corrêa-Oliveira R. Cellular responses and cytokine production in post-treatment hookworm patients from an endemic area in Brazil. Clin Exp Immunol. 2004 May;136(2):334–40. doi: 10.1111/j.1365-2249.2004.02449.x 15086399 PMC1809034

[4] Geiger SM, Caldas IR, Mc Glone BE, Campi-Azevedo AC, De Oliveira LM, Brooker S et al. Stage-specific immune responses in human Necator americanus infection. Parasite Immunol. 2007 Jul;29(7):347–58. doi: 10.1111/j.1365-3024.2007.00950.x 17576364 PMC1976388

[5] Ricci ND, Fiúza JA, Bueno LL, Cançado GG, Gazzinelli-Guimarães PH, Martins VG et al. Induction of CD4(+)CD25(+)FOXP3(+) regulatory T cells during human hookworm infection modulates antigen-mediated lymphocyte proliferation. PLoS Negl Trop Dis. 2011 Nov;5(11):e1383. doi: 10.1371/journal.pntd.0001383 22087344 PMC3210756

[6] Navarro S, Pickering DA, Ferreira IB, Jones L, Ryan S, Troy S, et al. Hookworm recombinant protein promotes regulatory T cell responses that suppress experimental asthma. Sci Transl Med. 2016 Oct 26;8(362):362ra143 doi: 10.1126/scitranslmed.aaf8807 27797959

[7] Sakaguchi S. Naturally arising Foxp3-expressing CD25+CD4+ regulatory T cells in immunological tolerance to self and non-self. Nat Immunol. 2005 Apr;6(4):345–52. doi: 10.1038/ni1178 15785760

[8] Miyara M, Yoshioka Y, Kitoh A, Shima T, Wing K, Niwa A et al. Functional delineation and differentiation dynamics of human CD4+ T cells expressing the FoxP3 transcription factor. Immunity. 2009 Jun 19;30(6):899–911. doi: 10.1016/j.immuni.2009.03.019 19464196

[9] Simonetta F, Lecuroux C, Girault I, Goujard C, Sinet M, Lambotte O et al. Early and long-lasting alteration of effector CD45RA(-)Foxp3(high) regulatory T-cell homeostasis during HIV infection. J Infect Dis. 2012 May 15;205(10):1510–9. doi: 10.1093/infdis/jis235 22457280 PMC3989210

[10] Deaglio S, Dwyer KM, Gao W, Friedman D, Usheva A, Erat A et al. Adenosine generation catalyzed by CD39 and CD73 expressed on regulatory T cells mediates immune suppression. J Exp Med. 2007 Jun 11; 204(6):1257–65. doi: 10.1084/jem.20062512 17502665 PMC2118603

[11] Takenaka MC, Robson S, Quintana FJ. Regulation of the T cell response by CD39. Trends Immunol 2016 37: 427–39. doi: 10.1016/j.it.2016.04.009 27236363 PMC5215082

[12] Lange C, Scholl M, Melms A, Bischof F. CD62L(high) Treg cells with superior immunosuppressive properties accumulate within the CNS during remissions of EAE. Brain Behav Immun. 2011 Jan;25(1):120–6. doi: 10.1016/j.bbi.2010.09.004 20837133

[13] Lu SY, Liu KY, Liu DH, Xu LP, Huang XJ. High frequencies of CD62L+ naive regulatory T cells in allografts are associated with a low risk of acute graft‐versus‐host disease following unmanipulated allogeneic haematopoietic stem cell transplantation. Clin Exp Immunol 2011;165:264–277. doi: 10.1111/j.1365-2249.2011.04418.x 21635226 PMC3142651

[14] Ermann J, Hoffmann P, Edinger M, Dutt S, Blankenberg FG, Higgins JP et al. Only the CD62L+ subpopulation of CD4+CD25+ regulatory T cells protects from lethal acute GVHD. Blood. 2005 Mar 1;105(5):2220–6. doi: 10.1182/blood-2004-05-2044 15546950

[15] Fu S, Yopp AC, Mao X, Chen D, Zhang N, Chen D, et al. CD4+ CD25+ CD62+ T-regulatory cell subset has optimal suppressive and proliferative potential. Am J Transplant. 2004 Jan;4(1):65–78. doi: 10.1046/j.1600-6143.2003.00293.x . 14678036

[16] Busse M, Krech M, Meyer-Bahlburg A, Hennig C, Hansen G. ICOS mediates the generation and function of CD4+CD25+Foxp3+ regulatory T cells conveying respiratory tolerance. J Immunol. 2012 Aug 15;189(4):1975–82. doi: 10.4049/jimmunol.1103581 22815292

[17] Li DY, Xiong XZ. ICOS+ Tregs: A Functional Subset of Tregs in Immune Diseases. Front Immunol. 2020 Aug 28;11:2104 doi: 10.3389/fimmu.2020.02104 32983168 PMC7485335

[18] Vocanson M, Rozieres A, Hennino A, Poyet G, Gaillard V, Renaudineau S et al. Inducible costimulator (ICOS) is a marker for highly suppressive antigen-specific T cells sharing features of TH17/TH1 and regulatory T cells. J Allergy Clin Immunol. 2010 Aug;126(2):280–9 doi: 10.1016/j.jaci.2010.05.022 20624644

[19] Agata Y, Kawasaki A, Nishimura H, Ishida Y, Tsubata T, Yagita H et al. Expression of the PD-1 antigen on the surface of stimulated mouse T and B lymphocytes. Int Immunol. 1996 May;8(5):765–72. doi: 10.1093/intimm/8.5.765 8671665

[20] Brown JA, Dorfman DM, Ma FR, Sullivan EL, Munoz O, Wood CR et al. Blockade of programmed death-1 ligands on dendritic cells enhances T cell activation and cytokine production. J Immunol. 2003 Feb 1;170(3):1257–66. doi: 10.4049/jimmunol.170.3.1257 12538684

[21] Thornton AM, Shevach EM. CD4+CD25+ immunoregulatory T cells suppress polyclonal T cell activation in vitro by inhibiting interleukin 2 production. J Exp Med. 1998 Jul 20;188(2):287–96. doi: 10.1084/jem.188.2.287 ; PMCID: PMC2212461. 9670041 PMC2212461

[22] Rubtsov YP, Rasmussen JP, Chi EY, Fontenot J, Castelli L, Ye X, et al. Regulatory T cell-derived interleukin-10 limits inflammation at environmental interfaces. Immunity. 2008 Apr;28(4):546–58. doi: 10.1016/j.immuni.2008.02.017 18387831

[23] Annacker O, Pimenta-Araujo R, Burlen-Defranoux O, Barbosa TC, Cumano A, Bandeira A. CD25+ CD4+ T cells regulate the expansion of peripheral CD4 T cells through the production of IL-10. J Immunol. 2001 Mar 1;166(5):3008–18. doi: 10.4049/jimmunol.166.5.3008 11207250

[24] Montresor A, Crompton, David W. T, Hall A, Bundy D. A. P, Savioli L. Guidelines for the evaluation of soil-transmitted helminthiasis and schistosomiasis at community level: a guide for managers of control programmes. World Health Organization. (‎1998)‎.

[25] Verbeke G, Molenberghs G. Linear Mixed Models for Longitudinal Data. Springer Series in Statistics. New-York: Springer-Verlag; 2000.

[26] https://www.who.int/news-room/fact-sheets/detail/soil-transmitted-helminth-infections

[27] Silver ZA, Kaliappan SP, Samuel P, Venugopal S, Kang G, Sarkar R et al. Geographical distribution of soil transmitted helminths and the effects of community type in South Asia and South East Asia—A systematic review. PLoS Negl Trop Dis. 2018 Jan 18;12(1):e0006153. doi: 10.1371/journal.pntd.0006153 29346440 PMC5773013

[28] Hung BK, De NV, Duyet le V, Chai JY. Prevalence of Soil-Transmitted Helminths and Molecular Clarification of Hookworm Species in Ethnic Ede Primary Schoolchildren in Dak Lak Province, Southern Vietnam. Korean J Parasitol. 2016 Aug;54(4):471–6. doi: 10.3347/kjp.2016.54.4.471 27658599 PMC5040093

[29] Mihrshahi S, Casey GJ, Montresor A, Phuc TQ, Thach DT, Tien NT et al. The effectiveness of 4 monthly albendazole treatment in the reduction of soil-transmitted helminth infections in women of reproductive age in Viet Nam. Int J Parasitol. 2009 Jul 15;39(9):1037–43. doi: 10.1016/j.ijpara.2009.01.013 19324046 PMC5621623

[30] WHO Expert Committee. Prevention and control of schistosomiasis and soil-transmitted helminthiasis. World Health Organ Tech Rep Ser. 2002;912:i–vi, 1–57, back cover. 12592987

[31] Chami GF, Fenwick A, Bulte E, Kontoleon AA, Kabatereine NB, Tukahebwa EM, et al. Influence of Schistosoma mansoni and Hookworm Infection. Intensities on Anaemia in Ugandan Villages. PLoS Negl Trop Dis. 2015 Oct 29;9(10):e0004193. Erratum in: PLoS Negl Trop Dis. 2015 Dec;9(12):e0004287. PMCID: PMC4626098. doi: 10.1371/journal.pntd.0004193 26513151 PMC4626098

[32] Forrer A, Vounatsou P, Sayasone S, Vonghachack Y, Bouakhasith D, Utzinger J, et al. Risk profiling of hookworm infection and intensity in southern Lao People’s Democratic Republic using Bayesian models. PLoS Negl Trop Dis. 2015 Mar 30;9(3):e0003486. doi: 10.1371/journal.pntd.0003486 25822794 PMC4378892

[33] Pritchard DI, Walsh EA, Quinell RJ, Raiko A, Edmonds P, Keymer AE. Isotypic variation in antibody responses in a community in Papua New Guinea to larval and adult antigens during infection, and following reinfection, with the hookworm Necator americanus. Parasite Immunol. 1992 Nov;14(6):617–31. doi: 10.1111/j.1365-3024.1992.tb00034.x . 1470481

[34] Nguyen T, Cheong FW, Liew JW, Lau YL. Seroprevalence of fascioliasis, toxocariasis, strongyloidiasis and cysticercosis in blood samples diagnosed in Medic Medical Center Laboratory, Ho Chi Minh City, Vietnam in 2012. Parasit Vectors. 2016 Sep 5;9(1):486. doi: 10.1186/s13071-016-1780-2 27595647 PMC5011968

[35] Romero Núñez C, Mendoza Martínez GD, Yañez Arteaga S, Ponce Macotela M, Bustamante Montes P, Ramírez Durán N. Prevalence and risk factors associated with Toxocara canis infection in children. ScientificWorldJournal. 2013 Jun 9;2013:572089. doi: 10.1155/2013/572089 23844404 PMC3690266

[36] Amoani B, Adu B, Frempong MT, Sarkodie-Addo T, Nuvor SV, Wilson MD, et al. Levels of serum eosinophil cationic protein are associated with hookworm infection and intensity in endemic communities in Ghana. PLoS One. 2019 Sep 12;14(9):e0222382. doi: 10.1371/journal.pone.0222382 31513658 PMC6742367

[37] Insiripong S and Kitsuntisumpun S. Eosinophil Count in Strongyloides, Hookworm, Liver Fluke or Taenia spp. Infestation. Trop Med Surg 2013, 1:3

[38] Klion AD, Nutman TB. The role of eosinophils in host defense against helminth parasites. J Allergy Clin Immunol. 2004 Jan;113(1):30–7. doi: 10.1016/j.jaci.2003.10.050 14713904

[39] Brooker S, Jardim-Botelho A, Quinnell RJ, Geiger SM, Caldas IR, Fleming F et al. Age-related changes in hookworm infection, anaemia and iron deficiency in an area of high Necator americanus hookworm transmission in south-eastern Brazil. Trans R Soc Trop Med Hyg. 2007 Feb;101(2):146–54 doi: 10.1016/j.trstmh.2006.05.012 17027054

[40] Maizels RM, Smith KA. Regulatory T cells in infection. Adv Immunol. 2011;112:73–136. doi: 10.1016/B978-0-12-387827-4.00003-6 22118407 PMC7150045

[41] Maizels RM, McSorley HJ. Regulation of the host immune system by helminth parasites. J Allergy Clin Immunol. 2016 Sep;138(3):666–675. doi: 10.1016/j.jaci.2016.07.007 27476889 PMC5010150

[42] Chang LY, Lin YC, Chiang JM, Mahalingam J, Su SH, Huang CT, et al. Blockade of TNF-α signaling benefits cancer therapy by suppressing effector regulatory T cell expansion. Oncoimmunology. 2015 Apr 16;4(10):e1040215 doi: 10.1080/2162402X.2015.1040215 26451304 PMC4589045

[43] Nafady-Hego H, Li Y, Ohe H, Zhao X, Satoda N, Sakaguchi S et al. The generation of donor-specific CD4+CD25++CD45RA+ naive regulatory T cells in operationally tolerant patients after pediatric living-donor liver transplantation. Transplantation. 2010 Dec 27;90(12):1547–55. doi: 10.1097/TP.0b013e3181f9960d 21085066

[44] Jaiswal S, Prakash Bhakuni, Priyanka Bharadwaj, Aby Joy, Aditi Chakrabarti, Shamsuz Zaman et al. CD45RA+ Regulatory T Cells (Tregs) in the Graft is Inversely Related to Donor Age and Impacts Early Alloreactivity and Survival in Younger Patients Undergoing Haploidentical PBSC Transplantation with Post-Transplantation Cyclophosphamide (PTCy). Biology of Blood and Marrow Transplantation 2018, Volume 24, Issue 3, S88—S89

[45] Lima NF, Gonçalves-Lopes RM, Kruize YCM, Yazdanbakhsh M, Ferreira MU. CD39 and immune regulation in a chronic helminth infection: The puzzling case of Mansonella ozzardi. PLoS Negl Trop Dis. 2018 Mar 5;12(3):e0006327. 10.1371/journal.pntd.0006327 PMC5854421 29505582

[46] Redpath SA, van der Werf N, Cervera AM, MacDonald AS, Gray D, Maizels RM et al. ICOS controls Foxp3(+) regulatory T-cell expansion, maintenance and IL-10 production during helminth infection. Eur J Immunol. 2013 Mar;43(3):705–15. doi: 10.1002/eji.201242794 23319295 PMC3615169

[47] Clay BS, Shilling RA, Bandukwala HS, Moore TV, Cannon JL, Welcher AA et al. Inducible costimulator expression regulates the magnitude of Th2-mediated airway inflammation by regulating the number of Th2 cells. PLoS One. 2009 Nov 4;4(11):e7525 doi: 10.1371/journal.pone.0007525 19888475 PMC2768787

[48] Vieira PL, Wassink L, Smith LM, Nam S, Kingsbury GA, Gutierrez-Ramos JC et al. ICOS-mediated signaling regulates cytokine production by human T cells and provides a unique signal to selectively control the clonal expansion of Th2 helper cells. Eur J Immunol. 2004 May;34(5):1282–90. doi: 10.1002/eji.200324417 15114661

[49] Raimondi G, Shufesky WJ, Tokita D, Morelli AE, Thomson AW. Regulated compartmentalization of programmed cell death-1 discriminates CD4+CD25+ resting regulatory T cells from activated T cells. J Immunol. 2006 Mar 1;176(5):2808–16. doi: 10.4049/jimmunol.176.5.2808 16493037

[50] Gaze S, Bethony JM, Periago MV. Immunology of experimental and natural human hookworm infection. Parasite Immunol. 2014 Aug;36(8):358–66. doi: 10.1111/pim.12088 . 25337625

[51] Quinnell RJ, Woolhouse ME, Walsh EA, Pritchard DI. Immunoepidemiology of human necatoriasis: correlations between antibody responses and parasite burdens. Parasite Immunol. 1995 Jun;17(6):313–8. doi: 10.1111/j.1365-3024.1995.tb00897.x . 7494644

[52] Geiger SM, Fujiwara RT, Freitas PA, Massara CL, Dos Santos Carvalho O, Corrêa-Oliveira R et al. Excretory-secretory products from hookworm l(3) and adult worms suppress proinflammatory cytokines in infected individuals. J Parasitol Res. 2011;2011:512154. doi: 10.1155/2011/512154 21772981 PMC3135091

[53] Chevalier MF, Didier C, Petitjean G, Karmochkine M, Girard PM, Barré-Sinoussi F et al. Phenotype alterations in regulatory T-cell subsets in primary HIV infection and identification of Tr1-like cells as the main interleukin 10-producing CD4+ T cells. J Infect Dis. 2015 Mar 1;211(5):769–79. doi: 10.1093/infdis/jiu549 25281758

[54] Cop N, Ebo DG, Bridts CH, Elst J, Hagendorens MM, Mertens C, et al. Influence of IL-6, IL-33, and TNF-α on human mast cell activation: Lessons from single cell analysis by flow cytometry. Cytometry B Clin Cytom. 2018 May;94(3):405–411. doi: 10.1002/cyto.b.21547 28802100

[55] Gubernatorova EO, Gorshkova EA, Namakanova OA, Zvartsev RV, Hidalgo J, Drutskaya MS, et al. Non-redundant Functions of IL-6 Produced by Macrophages and Dendritic Cells in Allergic Airway Inflammation. Front Immunol. 2018 Nov 26;9:2718. doi: 10.3389/fimmu.2018.02718 30534125 PMC6276801

[56] Plitas G, Rudensky AY. Regulatory T Cells: Differentiation and Function. Cancer Immunol Res. 2016 Sep 2;4(9):721–5 doi: 10.1158/2326-6066.CIR-16-0193 27590281 PMC5026325

[57] Giacomin P, Zakrzewski M, Croese J, Su X, Sotillo J, McCann L et al. Experimental hookworm infection and escalating gluten challenges are associated with increased microbial richness in celiac subjects. Sci Rep. 2015 Sep 18;5:13797 doi: 10.1038/srep13797 26381211 PMC4585380

[58] Zaiss MM, Harris NL. Interactions between the intestinal microbiome and helminth parasites. Parasite Immunol. 2016 Jan;38(1):5–11. doi: 10.1111/pim.12274 26345715 PMC5019230

[59] McKay DM, Shute A, Lopes F. Helminths and intestinal barrier function. Tissue Barriers. 2017 Jan 2;5(1):e1283385 doi: 10.1080/21688370.2017.1283385 28452686 PMC5362995

[60] Metenou S, Coulibaly YI, Sturdevant D, et al. Highly heterogeneous, activated, and short-lived regulatory T cells during chronic filarial infection. *Eur J Immunol*. 2014;44(7):2036–2047. doi: 10.1002/eji.201444452 24737144 PMC4809647

[61] Flohr C, Tuyen LN, Quinnell RJ, Lewis S, Minh TT, Campbell J et al. Reduced helminth burden increases allergen skin sensitization but not clinical allergy: a randomized, double-blind, placebo-controlled trial in Vietnam. Clin Exp Allergy. 2010 Jan;40(1):131–42. doi: 10.1111/j.1365-2222.2009.03346.x 19758373

