# Supplementary figures and images for: Hookworm treatment induces a decrease of suppressive regulatory T cell associated with a Th2 inflammatory response
Source: PLoS One. 2021 Jun 10;16(6):e0252921. doi: 10.1371/journal.pone.0252921 (PMC8191899; doi:10.1371/journal.pone.0252921)

S1 Fig: Activated CD4+CD25hiFoxP3- lymphocytes

***


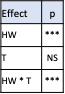

Supplement: S1 Fig — HW+: hookworm infected subjects, HW-: control subjects. 2W: 2 weeks, 1M: 1 month, 3M: 3 months and 12M: 12 months after treatment. HW+ group is represented with black square with continuous lines and HW- group with a blank circle with discontinuous lines. To compare the HW+ and HW- groups and their evolution, a maximum likelihood analysis has been used to test a group (HW), a time (T) and a group * time (HW * T) interaction effects (with data at T0, 2W and 1M). Statistical results are summarized in correspondent tables for each parameter. To compare longitudinal data from the HW+ group (T0, 2W, 1M, 3M and 12M), a time and a time2 effects were tested. The time effect is depicted on the figures and the time2 effect is given in the results section (* p<0.05, ** p<0.01, ***p<0.001). (DOCX) [file pone.0252921.s001.docx]
