# Supplementary material for: Hookworm treatment induces a decrease of suppressive regulatory T cell associated with a Th2 inflammatory response
Source: PLoS One. 2021 Jun 10;16(6):e0252921. doi: 10.1371/journal.pone.0252921 (PMC8191899; doi:10.1371/journal.pone.0252921)
Supplement: S1 Table — (DOCX) [file pone.0252921.s002.docx]

S1 Table: Antibodies used in flow cytometry experiments

| **Marker** | **Fluorochrome** | **Clone** | **Catalog numbers** | **Volume** |
| --- | --- | --- | --- | --- |
| CD4 | APC-H7 | RPA-T4 | 560158 | 5μL |
| CD25 | PE-Cy™7 | M-A251 | 557741 | 5μL |
| FoxP3 | Alexa Fluor® 488 | 259D/C7 | 560047 | 20μL |
| CD279 (PD-1) | PerCP-Cy™5.5 | EH12.1 | 561273 | 5μL |
| CD278 (ICOS) | PE | DX29 | 557802 | 20μL |
| CD62L | PE | DREG-56 | 555544 | 20μL |
| CD45RA | PerCP-Cy™5.5 | HI100 | 563429 | 5μL |
| CD39 | APC | TU66 | 560239 | 20μL |
