# Supplementary material for: Hookworm treatment induces a decrease of suppressive regulatory T cell associated with a Th2 inflammatory response
Source: PLoS One. 2021 Jun 10;16(6):e0252921. doi: 10.1371/journal.pone.0252921 (PMC8191899; doi:10.1371/journal.pone.0252921)
Supplement: S2 Table — (✻): Medical history: HW+: stroke, steatosis 2, hypertension, hepatitis B; HW-: hypertension 3, digestive bleeding, dengue, lithiasis, valvulopathy, Basedow disease. Comparison of proportion was tested with Pearson Chi-square test and comparison of mean with t-test. (* p<0.05, ** p<0.01, ***p<0.001). (DOCX) [file pone.0252921.s003.docx]

S2 Table

| Variable | HW-  (n=14) | HW+  (n=20) | p-value |
| --- | --- | --- | --- |
| Gender (M) | 8/14 (57.14%) | 14/20 (70%) | NS |
| Age | 42.14 ± 11.22 | 47.85 ± 10.21 | NS |
| Ethnic (kinh) | 14/14 (100%) | 20/20 (100%) | / |
| Years in Hoc Mon (Mean ± SD) | 42 [22.5 – 47.75] | 46 [42 – 57] | NS |
| Occupation (outside) | 5/14 | 18/20 | p<0.001 |
| Pets | 11/14 (74.57%) | 15/20 (75%) | NS |
| Garden | 6/14 (42.86%) | 18/20 (90%) | p<0.001 |
| Concrete house | 14/14 (100%) | 20/20 (100%) | / |
| Latrine system | 12/14 (85.71%) | 20/20 (100%) | NS |
| BMI (kg/m^2^) | 24.17 [20.91 – 26.20] | 20.29 [19.41 – 21.83] | p<0.05 |
| Parasite infection history | 1 | 0 | NS |
| Medical history (✻) | 7/14 (50%) | 11/20 (55%) | NS |
