# Supplementary material for: Hookworm treatment induces a decrease of suppressive regulatory T cell associated with a Th2 inflammatory response
Source: PLoS One. 2021 Jun 10;16(6):e0252921. doi: 10.1371/journal.pone.0252921 (PMC8191899; doi:10.1371/journal.pone.0252921)
Supplement: S3 Table — Results are expressed as mean +/- standard deviation. WBC: white blood count, N: Neutrophils, Eo: Eosinophils, Ly: Lymphocytes, Hb: Hemoglobin, MCV: Mean corpuscular volume, Alb: Albumin, NS: Non-significant. Maximum likelihood (ML) analysis on data from the 2 groups (at T0, 2W and 1M) were performed: a group effect, a time effect and a group * time interaction effect were tested. The time and time-square effects were tested on the longitudinal date from the HW+ group (ML 1 group). A positive time effect indicates an increase throughout the study and negative time-squared effect a decreased at the end of the study (and inversely). (DOCX) [file pone.0252921.s004.docx]

S3 Table

|  | **HW+** | | | | | **HW-** | | | **ML (2 Groups)** | | | **ML**  **(1 Group)** | |
| --- | --- | --- | --- | --- | --- | --- | --- | --- | --- | --- | --- | --- | --- |
|  | T0 | 2W | 1M | 3M | 12M | T0 | 2W | 1M | Time | HW | HW * Time | Time | Time^2^ |
| WBC (G/L) | 8002.7 ± 1845.4 | 7706.4 ± 1420.3 | 7706.4 ± 1420.3 | 7706.4 ± 1420.3 | 7706.4 ± 1420.3 | 7598.5 ± 1982.3 | 7470.7 ± 2117.2 | 7513.8 ± 2079.4 | NS | NS | NS | NS | NS |
| N (%) | 50.2 ± 11.4 | 56.5 ± 8.5 | 52.2 ± 8.1 | 52.2 ± 7.4 | 57.9 ± 12.7 | 57.1 ± 6.6 | 58.2 ± 6.6 | 56.2 ± 7.7 | NS | p<0.05 | NS | NS | NS |
| Eo (%) | 7.4 ± 5.3 | 4.5 ± 3.3 | 6.0 ± 3.9 | 5.8 ± 5.1 | 4.7 ± 4.2 | 4.4 ± 3.3 | 4.0 ± 2.7 | 3.7 ± 2.2 | NS | p<0.05 | NS | p<0.05  (-) | NS |
| Ly (%) | 34.6 ± 7.3 | 31.7 ± 6.4 | 34.5 ± 6.8 | 34.1 ± 5.2 | 30.4 ± 10.2 | 30.7 ± 6.3 | 30.7 ± 5.3 | 32.5 ± 7.4 | NS | NS | NS | NS | NS |
| N (G/L) | 4377.7 ± 1786.9 | 4376.8 ± 1096.9 | 3995.1 ± 1142.4 | 3993.5 ± 1126.2 | 5469.4 ± 2746.4 | 4367.2 ± 1289.4 | 4399.8 ± 1482.8 | 4171.8 ± 1074.8 | NS | NS | NS | NS | NS |
| Eo (G/L) | 549.0 ± 452.1 | 356.9 ± 282.6 | 459.6 ± 297.8 | 476.6 ± 486.7 | 397.8 ± 327.7 | 336.9 ± 277.4 | 311.3 ± 278.6 | 289.4 ± 208.7 | NS | NS | NS | p<0.05  (-) | p<0.05  (+) |
| Ly (G/L) | 2725.8 ± 607.0 | 2426.5 ± 616.2 | 2590.1 ± 700.0 | 2554.7 ± 529.4 | 2550.2 ± 569.6 | 2330.0 ± 801.6 | 2251.9 ± 609.7 | 2494.1 ± 1140.3 | NS | NS | NS | NS | NS |
| Hb (g/L) | 13.9± 1.5 | 13.8 ± 1.9 | 13.7 ± 1.7 | 14.0 ± 1.7 | 14.4± 3.3 | 14.4 ± 1.5 | 14.2 ± 1.1 | 14.2 ± 1.1 | NS | NS | NS | NS | NS |
| MCV (µm3) | 90.5 ± 9.5 | 92.7 ± 9.1 | 92.4 ± 9.9 | 91.4 ± 9.7 | 95.4 ± 7.5 | 83.2 ± 7.5 | 88.2 ± 7.4 | 85.0 ± 7.3 | NS | NS | NS | NS | NS |
| Ferritin (ng/mL) | 217.6±189.6 | - | - | 199±240.9 | 300.9±246.6 | 281.6±312.2 | - | - | NS | NS | NS | NS | NS |
| Alb (g/L) | 44.8 ± 3.3 | 45.0 ± 3.7 | 45.7 ± 3.3 | 46.8± 2.8 | 44.4± 6.4 | 45.1 ± 1.8 | 45.6± 4.7 | 45.7 ± 1.7 | NS | NS | NS | p<0.01 (+) | p<0.01  (-) |
